# Lymphangiogenic Markers and Their Impact on Nodal Metastasis and Survival in Non-Small Cell Lung Cancer - A Structured Review with Meta-Analysis

**DOI:** 10.1371/journal.pone.0132481

**Published:** 2015-08-25

**Authors:** Thomas K. Kilvaer, Erna-Elise Paulsen, Sigurd M. Hald, Tom Wilsgaard, Roy M. Bremnes, Lill-Tove Busund, Tom Donnem

**Affiliations:** 1 Department of Oncology, University Hospital of North Norway, Tromso, Norway; 2 Institute of Clinical Medicine, UiT The Arctic University of Norway, Tromso, Norway; 3 Department of Community Medicine, UiT The Arctic University of Norway, Tromso, Norway; 4 Department of Clinical Pathology, University Hospital of North Norway, Tromso, Norway; 5 Institute of Medical Biology, UiT The Arctic University of Norway, Tromso, Norway; The University of Hong Kong, CHINA

## Abstract

**Background:**

In non-small cell lung cancer (NSCLC), nodal metastasis is an adverse prognostic factor. Several mediating factors have been implied in the development of nodal metastases and investigated for predictive and prognostic properties in NSCLC. However, study results differ. In this structured review and meta-analysis we explore the published literature on commonly recognized pathways for molecular regulation of lymphatic metastasis in NSCLC.

**Methods:**

A structured PubMed search was conducted for papers reporting on the expression of known markers of lymhangiogenesis in NSCLC patients. Papers of sufficient quality, presenting survival and/or correlation data were included.

**Results:**

High levels of vascular endothelial growth factor C (VEGF-C, HR 1.57 95% CI 1.34–1.84) and high lymphatic vascular density (LVD, HR 1.84 95% CI 1.18–2.87) were significant prognostic markers of poor survival and high expression of VEGF-C, vascular endothelial growth factor receptor 3 (VEGFR3) and LVD was associated with lymph node metastasis in NSCLC.

**Conclusion:**

Lymphangiogenic markers are prognosticators of survival and correlate with lymph node metastasis in NSCLC. Their exact role and clinical implications should be further elucidated.

## Introduction

Lung cancer is estimated to have the second highest incidence of all cancers in US women and men [1]. With a dismal prognosis, and over 163 000 expected deaths in 2014, lung cancer is the number one killer amongst cancers [1]. Lung cancer is staged according to the system advocated by the American Joint Committee on Cancer (AJCC) and the Union Internationale Contre le Cancer (UICC) which as of 2010 is in its 7^th^ edition [2,3]. The system is built around tumor size and localization (T), extent of nodal involvement (N) and presence of distant metastasis (M) [3]. In short, stage I comprises small localized tumors, while stages II-IIIA represent larger tumors with or without nodal metastasis [3]. The five-year-survival for stage IA, IB, IIA, IIB and IIIA lung cancer is 65–81%, 54–72%, 46–59%, 46–47% and 33–38% respectively [4–6]. Stage IIIB and IV lung cancers are not considered for surgery and have an abysmal prognosis [4–6]. Non-small cell lung cancer (NSCLC) represent >80% of all lung cancer cases [7].

Nodal metastasis represents a major shift in NSCLC biology, from a localized to an invasive phenotype. Patients presenting with nodal metastases have a more advanced stage and a worse prognosis compared to patients without nodal involvement and the same tumor size [3]. Further, the prognosis of patients radically operated for stage I NSCLC differs widely, with 20–35% of patients developing recurrent disease, often in localized lymph nodes [4–6,8]. Vascular endothelial growth factors (VEGF) -C and -D, and their corresponding receptor vascular endothelial growth factor receptor 3 (VEGFR3, also known as Flt4), are by many considered the main players in the development of tumor associated lymphatic vessels [9]. These work by recruiting endothelial cells (ECs) and other stromal cells to develop and maintain a crude lymphatic network in the tumor micro-environment [9,10]. In tumor models of NSCLC, the presence of VEGF-C and VEGFR3 leads to proliferation, invasiveness and nodal metastases [11]. There is also evidence supporting that tumor derived VEGF-C induces the development of lymphatic vasculature in premetastatic lymph nodes; thus preparing them for the arrival and hosting of cancer cells [12,13]. The inhibition of VEGFR3 in a xenograft model of NSCLC indicated that abrogation of lymphangiogenesis could prevent lymphatic metastasis [14]. Interestingly, anti-VEGFR3 treatment had to be initiated before the development of lymphatic vasculature for its effect to take place and it did not abrogate lymphatic vessel co-option [14].

Lymphatic vessel density (LVD) represent the density of lymphatic vessels in the tumor micro-environment. For many cancers LVD is an established marker of adverse prognosis, but no real consensus regarding its evaluation exist [15–18]. A connection between tumor VEGF-C expression and LVD in sentinel lymph nodes has been suggested [19]. In NSCLC, the expression of VEGF-C [20–28], VEGF-D [21,22,29] and VEGFR3 [30] as well as lymphatic vessel density (LVD) [21,23,28,31–33] have been correlated to nodal metastasis and linked to patient survival. Intriguingly, other studies fail to show these relationships.

Herein we present a structured review and meta-analysis of lymphangiogenesis and its relationship with lymph node metastasis and survival in NSCLC with emphasis on VEGF-C, VEGF-D, VEGFR3 and LVD.

## Materials and Methods

### Search strategy and study selection

The electronic database MEDLINE was searched for studies reported up to a date limit set to Sept. 22, 2014 with no lower date limit applied. The search string used for lymphangiogenesis was ((lymphatic vessel density) OR (LVD) OR (lymphatic micro vessel density) OR (LMVD) OR (D2-40) OR (VEGFR3) OR (flt4) OR (VEGF-C) OR (CD34)) AND ((non-small cell lung cancer) OR (non small lung cell cancer) OR (non-small lung cell cancer) OR (NSCLC) OR (adenocarcinoma of the lung) OR (lung adenocarcinoma) OR (squamous cell carcinoma of the lung) OR (lung squamous cell carcinoma) OR (lung SCC) OR (large-cell carcinoma of the lung) OR (lung large-cell carcinoma) OR (lung LCC)) AND (Humans[Mesh] AND English[lang]).

The searches were restricted to human species and English language. Publications selected for full reading were explored to complete the searches.

Inclusion criteria for the meta-analyses were as follows: (1) measure of VEGF-C, VEGF-D or VEGFR3 in primary NSCLC tissue using immunohistochemistry (IHC) or enzyme linked immunosorbent assay (ELISA)/reverse transcription-polymerase chain reaction (RT-PCR) or a measure of LVD; (2) providing survival information in the form of HR with 95% CI OR numbers with high/low expression and survival curves OR correlation with lymphatic metastasis and sufficient data to calculate RR; (3) follow-up exceeding two years; (4) only the most recent OR most adequate publication was used in the case of the same author reporting on the same population. Two reviewers (T. K and T. D) independently determined the study eligibility of publications selected for full reading, with disagreement resolved by consensus.

### Data extraction and quality assessment

The eligible studies were assessed by two independent reviewers (T. K and T. D). The data retrieved from each study included author(s), test method, cut-off and data on survival and/or lymphatic metastasis. In the case of missing data the author of the primary study was contacted using the email address supplied in the original publication, missing data not supplied by the authors were extracted from the summary statistics where possible.

To assess the overall quality of studies included in the meta-analysis, these were scored according to a modified quality scale for biological prognostic factors for lung cancer developed by Steels et al. for the European Lung Cancer Working Group [34]. The quality scale incorporates four dimensions of method assessment 1) Scientific design (5 questions); 2) Laboratory methodology (7 questions); 3) Generalizability (6 questions); 4) Results analysis (4 questions). All questions were scored using an ordinal scale (values: 2, 1, 0), with results given as percentages of achievable points within each category [34].

### Statistical analyses

All analyses were conducted in R-studio version 0.98.1087 using R-kernel 3.1.1.

For the meta-analyses of markers in relation to survival endpoints, univariate hazard ratios (HR) with corresponding 95% confidence intervals (CI) were combined to give the aggregated effect estimates. For cases in which these values were not reported, or supplied from the author(s) upon request, the data were extrapolated from available numerical data and survival curves according to methods described by Parmar et. al [35]. For cases in which survival curves were used, these were first digitalized using Engauge digitizer [36]. For the analysis of survival curves constant censoring during the follow-up of survivors were assumed. A 3-month interval between censoring calculations was deemed adequate. For papers with overall survival as end-point censoring was assumed to start at the time of minimum follow-up. For papers based on disease-specific survival censoring was assumed to start at patient inclusion. Maximal follow-up was set to the difference between minimum follow-up and the date of last follow-up. For papers where the date of the last follow-up was not given these dates were extrapolated from the survival curves.

For the meta-analyses of markers in relation to nodal metastasis, data was extracted in the form of 2x2 tables from which the effect estimates were calculated.

Aggregation of data was conducted using the R-package “metafor” [37]. A certain level of heterogeneity was expected and because of this, a random effects model was used to estimate the HRs and corresponding 95% CIs. Overall heterogeneity was examined using Q, tau ^2^, I^2^ and H^2^ statistics [38]. To explore the heterogeneity introduced by each study included in the meta-analyses, a function leaving out one article at a time (leave1out in the “metaphor” package) was used. Subgroup analyses, according to stage and histology, (adenocarcinoma or squamous cell carcinoma) were conducted for markers where sufficient information was provided.

Egger's test was used to evaluate publication bias [39]. Contour enhanced funnel plot's were used to help interpret, and to further explore publication bias in the case of funnel asymmetry [40]. The trim and fill method was used to visualize and to adjust for missing studies in the case of publication bias [41].

## Results

### Study selection and characteristics

**Fig 1:**
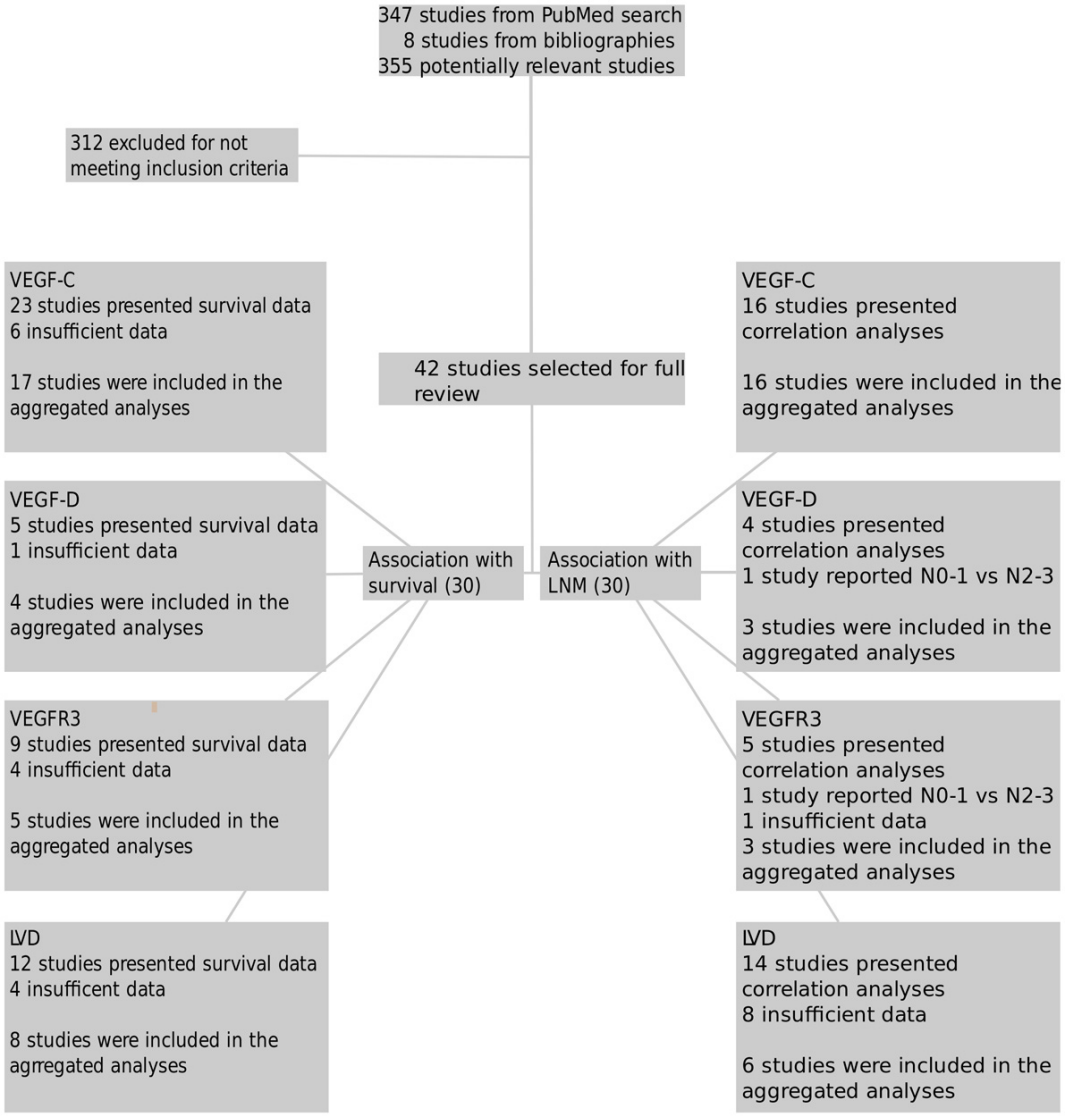
Illustration of the literature search and inclusion processes. Abbreviations: VEGF, vascular endothelial growth factor; VEGFR, vascular endothelial growth factor receptor; LVD, lymphatic vascular density.

Fig 1 summarizes the search strategy and inclusion processes. Three-hundred-and-forty-seven studies were identified in the initial search with an additional eight studies identified from reading bibliographies. After initial screening 42 studies were selected for full review [20–33,42–70]. The articles selected for full review and subsequently included in the meta-analyses are summarized in Table 1. As expected several papers reported on more than one lymphangiogenic marker.

**Table 1:** Summarizing the studies included in the meta-analyses.

Study	Year	N	Design	Histology	Stage	Method	Marker	Positive	Cut-off	Survival		LNM	QS	QS
										HRe	HR (95% CI)	RR (95% CI)	Surv	LNM
Adachi et al.[21]	2007	76	Retro	All	I-IIIb	IHC	VEGF-C	20%	10%			2.85(1.3–6.2)		0.54
							VEGF-D	36%	50%			3.33(1.4–8.0)		
							LVD	34%	≥20			4.81(2.1–10.9)		
Arinaga et al.[43]	2003	180	Retro	All	I-III	IHC	VEGF-C	76%	30%	Surv	1.52(0.8–2.5)		0.66	
							VEGFR3	22%	CS	Surv	2.37(1.5–3.2)			
Chen et al.[47]	2011	49	Retro	All	I	IHC	VEGF-C	49%	CS	Surv	0.94(0.0–426.2)		0.72	
Carilla de Santa et al.	2009	48	Retro	All	I-IV	IHC	VEGF-C	29%	CS	Surv	2.51(1.1–5.7)		0.67	
[44]							VEGF-D	23%	CS	Surv	0.55(0.2–1.8)			
							VEGFR3	42%	CS	Surv	0.54(0.2–1.3)			
Donnem et al.[30,70]	2007/	335	Retro	All	I-IIIa	IHC	VEGF-C	31%	CS	HR	1.30(0.9–1.8)	1.14(0.8–1.6)	0.83	0.8
	2009						VEGF-D	68%	CS	HR	1.27(0.9–1.9)	1.25(0.9–1.8)		
	*						VEGFR3	36%	CS	HR	1.69(1.2–2.4)	1.56(1.1–2.2)		
Enatsu et al.[48]	2006	78	Retro	Adeno	I-III	IHC	VEGF-C	42%	10%	HR	0.47(0.1–1.6)		0.58	
Faoro et.al.[49]	2008	77	Retro	All	I-IV	IHC	LVD	48%	Mean	Surv	0.88(0.5–1.7)		0.59	
Feng et al.[22]	2010	96	Retro	All	I-IIIa	IHC,	VEGF-C	44%	30%			1.41(0.9–2.2)		0.57
						RT-PCR	VEGF-D	24%	30%			0.93(0.6–1.6)		
Guo et al.[23]	2009	65	Retro	All	I-IV	IHC	VEGF-C	77%	CS			9.90(1.5–66.5)	0.64	0.59
							LVD	46%	28	Surv	1.97(1.0–3.8)	6.77(3.0–15.3)		
Huang et al.[50]	2005	97	Retro	All	I	IHC	VEGF-C	40%	30%	Surv	2.45(0.8–7.1)		0.75	
		76			II-III			45%			0.91(0.5–1.7)			
Iwakiri et al.[51]	2009	215	Retro	All	I-IIIa	IHC	LVD	51%	≥38	Surv	2.13(0.9–5.0)	1.05(0.7–1.5)	0.77	0.76
Kadota et al.[54]	2008	147	Retro	All	I-III	IHC	VEGF-C	44%	30%	HR	1.82(1.1–3.5)		0.79	
							LVD	44%	≥15	HR	1.77(1.0–3.1)			
Kajita et al.[24]	2001	62	Retro	All	I-IV	IHC	VEGF-C	39%	CS	Surv	2.53(0.9–7.1)	2.02(1.1–3.7)	0.59	0.55
Ko et al.[29]	2008	118	Retro	All	I-III	IHC	VEGF-C	60%	CS	Surv	1.21(0.6–2.5)		0.64	
							VEGF-D	53%	CS	Surv	1.11(0.5–2.4)			
Kojima et al.[55]	2005	129	Retro	All	I-IIIb	IHC	VEGF-C	43%	CS	HR	2.72(1.4–5.4)	1.83(0.9–3.8)	0.76	0.69
							VEGFR3	57%	CS	HR	3.04(1.5–6.7)	2.30(1.0–5.4)		
Li et al.[31]	2003	76	Retro	All	I-IV	IHC	VEGF-C	72%	10%	Surv	3.12(1.4–6.8)		0.47	
Li et al.[20]	2010	70	Retro	All	I-IIIb	IHC	VEGF-C	70%	CS			2.46(1.0–6.3)		0.61
Maekawa et al.[56]	2007	55	Retro	All	I	RT-PCR	VEGF-C	NG	Mean	HR	2.31(0.5–10.0)		0.52	
							VEGF-D	NG	Mean	HR	0.29(0.1–0.9)			
							VEGFR3	NG	Mean	HR	0.37(0.0–2.8)			
Min et al.[57]	2011	97	Retro	All	I-III	IHC	LVD	70%	≥6	Surv	0.49(0.1–1.9)	1.03(0.5–2.0)	0.65	0.62
Nakashima et al.[58]	2004	153	Retro	All	I-IIIb	IHC	VEGF-C	42%	30%	Surv	1.48(0.8–2.7)	0.98(0.6–1.4)	0.77	0.74
Ogawa et al.[61]	2004	206	Retro	All	I-IIIb	IHC	VEGF-C	61%	CS	Surv	1.37(0.8–2.3)	0.94(0.6–1.4)	0.65	0.58
Ohta et al.[62]	2000	122	Retro	All	I	IHC	VEGF-C	45%	CS	HR	1.34(0.6–3.1)	3.62(1.8–6.9)	0.68	0.63
Renyi-vamos et al.[63]	2005	103	Retro	All	I-IIIa	IHC	LVD	50%	Median	Surv	2.29(1.2–4.4)		0.69	0.64
							VEGF-C	56%	30%			0.99(0.7–1.5)		
Saintigny et al.[64]	2007	92	Prosp	All	I-III	IHC	VEGF-C	74%	CS			1.63(0.9–3.0)		0.69
							VEGFR3	42%	CS			1.86(1.2–2.8)		
Su et al.[65]	2004	59	Retro	Adeno	I-IV	IHC	VEGF-C	49%	CS	Surv	1.69(1.0–3.0)		0.59	
Sun et al.[66]	2009	82	Retro	All	I-IV	IHC	LVD	50%	≥19.9	Surv	1.54(0.7–3.3)		0.67	
Takanami et al.[33]	2006	77	Retro	All	I-IIIa	IHC	VEGF-C	59%	10%			2.70(1.3–5.8)		0.66
							LVD	49%	≤25			4.11(1.1–4.5)		
Yamashita et al.[69]	2010	117	Retro	All	I	IHC	VEGF-C	49%	10%	HR	1.89(1.0–3.5)		0.82	
Zhang et al.[28]	2011	65	Retro	Adeno	I-IV	IHC	LVD	49%%	≤10	Surv	4.91(3.4–7.1)		0.62	
Zuo et al.[27]	2008	48	Retro	All	I-III	IHC	VEGF-C	69%	CS			3.18(0.8–1.6)		0.48

Abbreviations: N, number; LNM, Lymphatic node metastasis; HR, hazard ratio; HRe, how the HR-estimate was obtained (HR = given in the text, Surv = estimated from survival curve); RR, relative risk; QS, quality score; CS, complex score; VEGF, vascular endothelial growth factor; VEGFR, vascular endothelial growth factor receptor; LVD, lymphatic vascular density; NG, not given.

Thirty studies reported survival data for VEGF-C (23), VEGF-D(4), VEGFR3(9) and LVD(13) [23,24,28,29,31,32,42–51,54–58,61–66,68–70]. Of these 17, four, five and eight studies included sufficient survival data and were included in the aggregated survival-analyses of VEGF-C [24,29,31,43,44,47,48,50,54–56,58,61,62,65,69,70] VEGF-D [29,44,56,70], VEGFR3 [43,44,55,56,70] and LVD [23,28,49,51,54,57,63,66] respectively, reporting on a total of 2185, 567, 825 and 849 patients.

Thirty studies reported correlations between nodal metastasis and VEGF-C (16), VEGF-D(4), VEGFR3(5) or LVD (14) [20–33,45,49–52,54–64,66,67]. Of these 16, three, three and six included sufficient data in the form of 2x2 tables to be included in the aggregated analyses of VEGF-C [20–24,27,30,33,50,55,58,61–64,66], VEGF-D [21,22,30], VEGFR3 [30,55,64] and LVD [21,23,33,51,54,57] respectively, reporting on a total of 1889, 507, 556 and 677 patients.

Of the 30 studies included in the analyses, 29 used IHC for marker evaluation, while one study used RT-PCR. The percentage of patients with positive markers and the cut-offs used for marker evaluation varied extensively between studies (Table 1) and markers. For the evaluation of IHC, some studies used a straight forward percentage of positive cells, while other studies used a complex score (CS) consisting of several traits, including, but not limited to, percentage of positive cells and staining intensity. For LVD the cut-offs ranged from ≥6 to ≥38 vessels per high power field.

None of the authors prompted for supplementary data responded to the request.

### Quality assessment

The scores from the quality assessment (QA) are given in Table 1 for the individual studies and summarized in Table 2 for the overall scores for each marker. The overall quality scores (QS) of papers reporting survival tended to be superior to papers reporting correlations with LNM. This difference was largest for the dimension "results analysis" and probably due to the fact that only one paper reporting associations with LNM explored their results in multivariable models.

**Table 2:** Results of the quality assessment of the included studies by marker. Maximal score for any given category is 1.

	Scientific	Laboratory	Generalizability	Results	Overall
	Design	Methodology		Analysis	Score
VEGF-C Survival	0.7(0.5–0.8)	0.6(0.4–0.8)	0.7(0.4–1)	0.7(0.5–1)	0.7(0.5–0.8)
VEGF-D Survival	0.7(0.7–0.8)	0.7(0.5–0.8)	0.8(0.6–1)	0.7(0.7–0.8)	0.7(0.6–0.8)
VEGFR3 Survival	0.7(0.7–0.8)	0.7(0.6–0.8)	0.8(0.6–1)	0.8(0.7–0.9)	0.7(0.7–0.8)
LVD Survival	0.7(0.7–0.8)	0.6(0.5–0.7)	0.7(0.6–1)	0.7(0.4–0.8)	0.7(0.6–0.8)
VEGF-C LNM	0.7(0.5–0.8)	0.6(0.5–0.8)	0.7(0.4–1)	0.5(0.3–0.7)	0.6(0.5–0.8)
VEGF-D LNM	0.7(0.6–0.8)	0.6(0.5–0.8)	0.7(0.5–1)	0.5(0.4–0.7)	0.6(0.5–0.8)
VEGFR3 LNM	0.8(0.7–0.8)	0.7(0.6–0.8)	0.9(0.8–1)	0.6(0.5–0.7)	0.7(0.7–0.8)
LVD LNM	0.7(0.6–0.8)	0.6(0.5–0.7)	0.7(0.5–1)	0.4(0.1–0.5)	0.7(0.5–0.8)

Abbreviations: LNM, Lymphatic node metastasis; VEGF, vascular endothelial growth factor; VEGFR, vascular endothelial growth factor receptor; LVD, lymphatic vascular density.

### Meta-analysis

**Fig 2:**
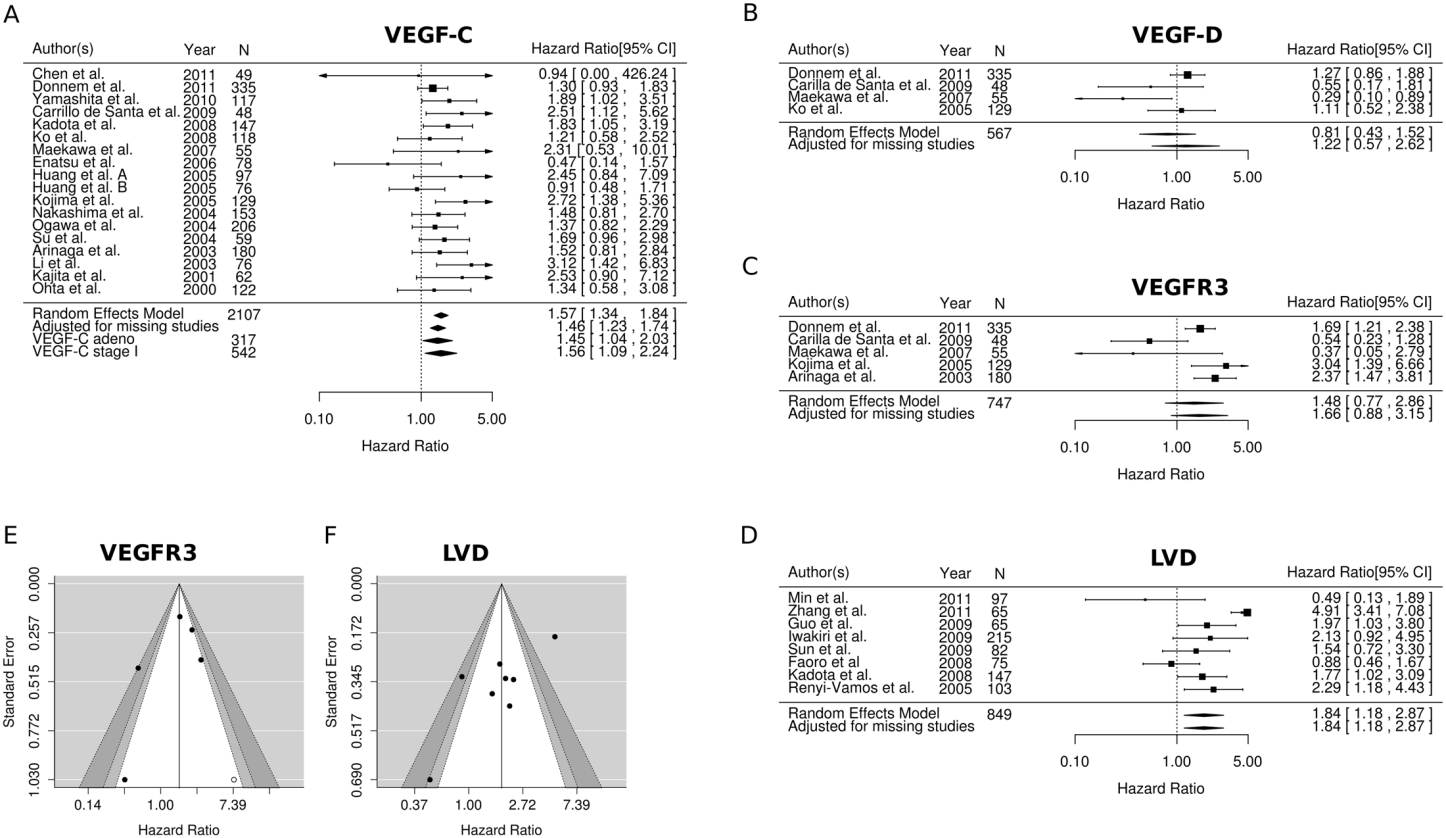
Forest plots of the survival meta-analyses for; A) VEGF-C, B) VEGF-D, C) VEGFR3, D) LVD, Funnel plots showing the relationship between the observed HR and the standard deviation in the survival meta-analyses for; E) VEGFR3, F) LVD. Abbreviations: VEGF, vascular endothelial growth factor; VEGFR, vascular endothelial growth factor receptor; LVD, lymphatic vascular density; N, number: HR, hazard ratio.

**Fig 3:**
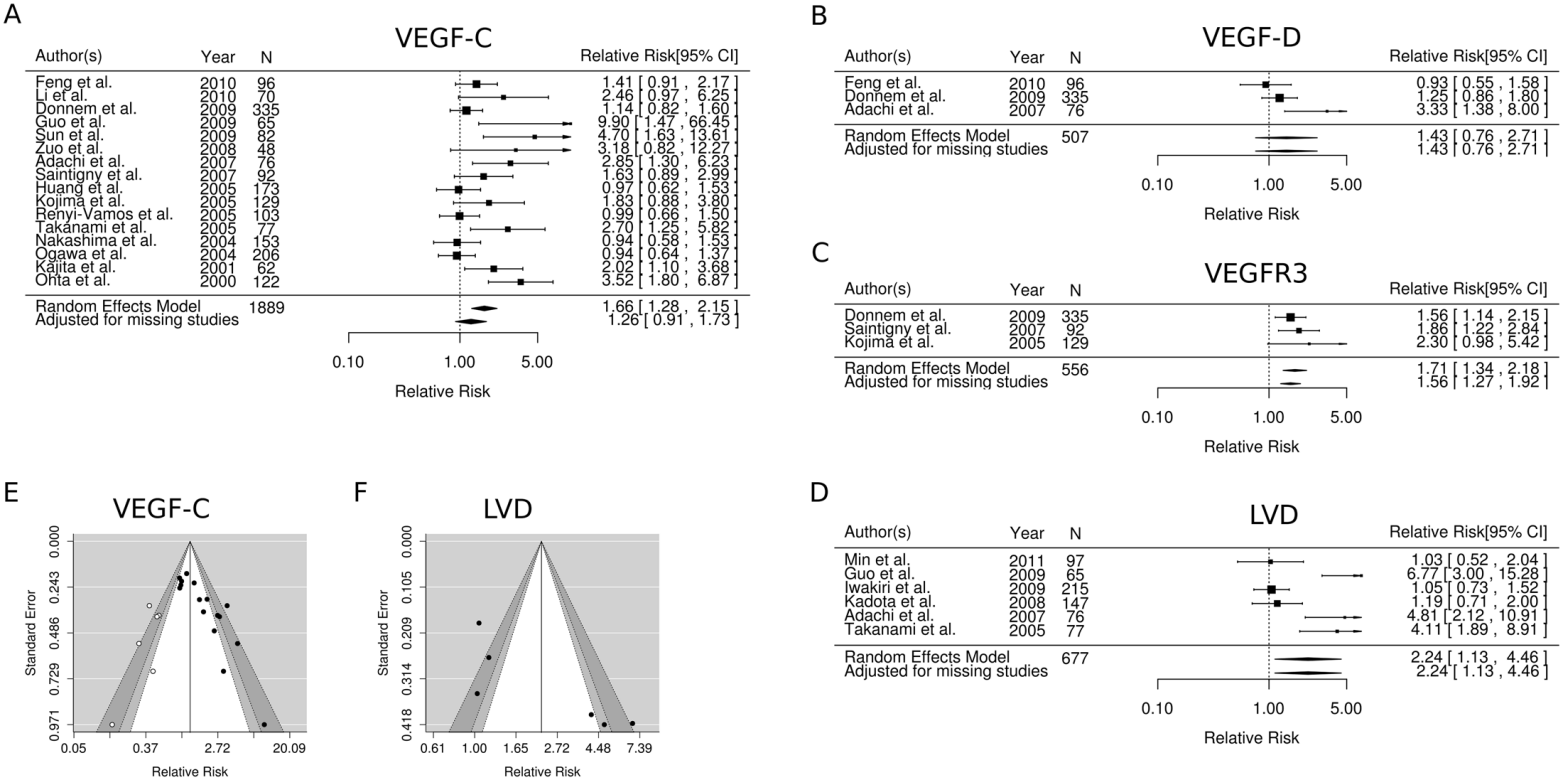
Forest plots of the lymphatic node metastasis meta-analyses for; A) VEGF-C, B) VEGF-D, C) VEGFR3, D) LVD, Funnel plots showing the relationship between the observed RR and the standard deviation in the lymphatic node metastasis meta-analyses for E) VEGF-C, F) LVD Abbreviations: VEGF, vascular endothelial growth factor; VEGFR, vascular endothelial growth factor receptor; LVD, lymphatic vascular density; N, number; RR, relative risk.

The results of the meta-analyses are summarized in Figs 2 and 3 and Table 3, while the individual univariate HRs, calculated or as reported, are given in Table 1.

**Table 3:** Summarizing the aggregated analyses and their corresponding heterogeneity tests. Values for unadjusted, leave1out (test conducted without the one study introducing the most heterogeneity) and adjusted (test conducted with the inclusion of studies assumed missing in case of funnel asymmetry).

	Survival summary						LNM summary					
	HR (95%CI)	Q	P	TAU^2^	I^2^(%)	H^2^	RR (95%CI)	Q	P	TAU^2^	I^2^(%)	H^2^
VEGF-C	1.57(1.34–1.84)	18.03	0.39	0	1.37	1.01	1.66(1.28–2.15)	40.47	<0.001	0.17	66.66	3
VEGF-C(leave1out)	1.62(1.38–1.91)	14.98	0.53	0	0	1	1.54(1.2–1.96)	32.52	<0.001	0.12	59.65	2.48
VEGF-C(adjusted)	1.46(1.23–1.73)	26.99	0.14	0.03	18.69	1.23	1.26(0.91–1.73)	76.53	<0.001	0.42	80.56	5.14
VEGF-D	0.81(0.43–1.52)	7.12	0.07	0.24	60.58	2.54	1.43(0.76–2.71)	5.96	0.05	0.23	74.49	3.92
VEGF-D(leave1out)	1.16(0.83–1.62)	1.73	0.42	0	0	1	1.13(0.84–1.53)	0.77	0.38	0	0	1
VEGF-D(adjusted)	1.22(0.57–2.62)	16.52	0.01	0.67	78.29	4.61	1.43(0.76–2.71)	5.96	0.05	0.23	74.49	3.92
VEGFR3	1.48(0.77–2.86)	12.88	0.01	0.39	79.07	4.78	1.71(1.34–2.18)	0.92	0.63	0	0	1
VEGFR3(leave1out)	1.98(1.46–2.68)	5.14	0.16	0.02	15.28	1.18	NA	NA	NA	NA	NA	NA
VEGFR3(adjusted)	1.66(0.88–3.15)	14.85	0.01	0.4	76.23	4.21	1.56(1.27–1.92)	2.87	0.58	0	0	1
LVD	1.84(1.18–2.87)	32.27	0	0.28	72.95	3.7	2.24(1.13–4.46)	32.52	<0.001	0.62	86.47	7.39
LVD(leave1out)	1.58(1.17–2.13)	8.38	0.21	0.03	18.24	1.22	2.67(1.25–5.72)	23.47	<0.001	0.62	82.73	5.79
LVD(adjusted)	NA	NA	NA	NA	NA	NA	2.24(1.13–4.46)	32.52	<0.001	0.62	86.47	7.39

Abbreviations: HR, hazard ratio; RR, relative risk; VEGF, vascular endothelial growth factor; VEGFR, vascular endothelial growth factor receptor; LVD, lymphatic vascular density; NA, not applicable; Q, test for heterogeneity; P, the p-value calculated for Q; TAU^2^, an estimate of the total amount of heterogeneity; I^2^, the proportion of total variation in study estimates attributed to heterogeneity; H^2^, the total variability or within study variance.

VEGF-C: The overall HR for survival in patients expressing high tumor cell VEGF-C was 1.57 (95% CI: 1.34–1.84) across 18 studies using a random effects model and including 2107 patients (Fig 2A, Table 3). When using the trim and fill method to adjust for missing studies the HR was 1.46 (95% CI: 1.23–1.73, Table 3). Subgroup analyses reporting on VEGF-C and survival in 4 studies of adenocarinomas (n = 317) and 5 studies of stage I NSCLC (n = 542) revealed HRs of 1.45 (95% CI: 1.04–2.03) and 1.56 (95% CI: 1.09–2.24), respectively (Fig 2A).

The overall RR for the association between VEGF-C and nodal metastasis was 1.66 (1.28–2.15) across 15 studies using a random effects model and including 1889 patients (Fig 3A, Table 3). When using the trim and fill method to adjust for possible missing studies the RR was 1.26 (0.91–1.73).

VEGF-D: In this meta-analysis VEGD-D was neither found to be associated with survival nor nodal metastasis in NSCLC patients.

VEGFR3: The overall HR for survival in patients expressing high tumor cell VEGFR3 was 1.48 (95% CI: 0.77–2.86) across 5 studies using a random effects model and including 747 patients (Fig 2C, Table 3). When using the trim and fill method to adjust for missing studies the HR was 1.66 (95% CI: 0.88–3.15, Table 3). Another approach, leaving out the one study introducing the most heterogeneity, revealed an overall HR for survival of 1.98 (95% CI: 1.46–2.68, Table 3).

The overall RR for association between VEGFR3 and nodal metastasis was 1.71 (95% CI: 1.34–2.18) across 3 studies and including 556 patients (Fig 3C, Table 3). When using the trim and fill method to adjust for possible missing studies the RR was 1.56 (95% CI: 1.27–1.92).

LVD: The overall HR for survival in patients expressing high levels of LVD in tumor was 1.84 (95% CI: 1.18–2.87) across 8 studies using a random effects model and including 849 patients (Fig 2D, Table 3). The trim and fill method did not suggest any missing studies and an adjusted HR was therefore not calculated.

The overall RR for the association between LVD and nodal metastasis was 2.24 (95% CI: 1.13–4.56) across 6 studies and including 667 patients (Fig 3D, Table 3). The trim and fill method did not suggest any missing studies and a adjusted RR was therefore not calculated.

### Publication bias

Egger's test for survival yielded p-values as follows: VEGF-C 0.400, VEGF-D 0.146, VEGFR3 0.020, VEGF-C in adenocarcinomas 0.073, VEGF-C in stage I patients 0.892 and LVD 0.006. This suggests significant publication bias in studies reporting on VEGFR3 and LVD and in the subgroup-analyses of VEGF-C. The contour enhanced Funnel plot for VEGFR3 (Fig 2E) suggests that the missing studies may be in an area of high statistical significance, but due to the low number of studies included, the Funnel plot has to interpreted with diligence. The contour enhanced Funnel plot for LVD (Fig 2F) suggests that the missing articles are those with low standard error of the effect estimate.

Egger's test for nodal metastasis yielded p-values as follows: VEGF-C <0.001, VEGF-D0.169, VEGFR3 0.375 and LVD 0.004. This suggests significant publication bias in the studies reporting on VEGF-C and LVD. The contour enhanced Funnel plot of VEGF-C (Fig 3E) indicates a strong positive publication bias. The contour enhanced Funnel plot of LVD (Fig 3F) indicates the lack of larger studies and a possible positive publication bias.

### Statistical considerations

The results of the aggregated analyses were obtained using the results reported in univariate analyses only. Some information can potentially be lost from studies reporting only multivariate results with inadequate data to reconstruct the univariate analysis. Multivariate data are only valid in their own multivariate system. Including multivariate data in the aggregated analysis provides increased numbers of patients at the cost of increased heterogeneity. This may be tolerated if all included studies are adequately large and includes the same, or approximately the same, variables in their multivariate models. In this meta-analysis the number of patients in the included studies ranged from 48 to 335 and the variables included in the different multivariate models varied (data not shown). Thus making the inclusion of data from multivariate analyses in the meta-analysis a dubious venture.

Heterogeneity is difficult to avoid when conducting meta-analyses based on marker data, as there exists no consensus on how such studies should be conducted. Among the included studies (Table 1), all but one were retrospective and all but one used IHC to detect the protein markers. Moreover, they differ largely with respect to selected antibodies, choice of cut-offs for the different markers and in the percentage of positive cases. The European Lung Cancer Working Party has proposed a quality scale for biological prognostic factors for lung cancer, but few studies adhere to this scale [34]. We utilized this scale to rate the included studies (Table 2). Mixed model approaches (data not shown) utilizing histology, stage, method, percentage of positives cases, cut-offs or quality scores as static modifiers were attempted, but none of these contributed to the overall model design and thus were rejected.

The present meta-analysis was restricted to articles published in the English language. This could introduce a positive selection bias as there is a tendency for positive studies to be published in English while negative studies more often are published in the authors native language [71]. Indeed, testing in the current meta-analyses suggested significant publication bias for studies reporting survival based on VEGFR3 and LVD and for LNM based on VEGF-C and LVD. Based on this the results of this meta-analysis have to be interpreted carefully and should be confirmed in larger trials.

Obtaining individual patient data for meta-analysis would theoretically help to define the role of lymphangiogenic markers by adjusting for the same confounders before data aggregation across all included studies [72]. However, this does not appear to be feasible as all authors were prompted by e-mail for additional information, but none of them replied.

## Discussion

### Summary of the meta-analyses

This is, to our knowledge, the most comprehensive structured review with meta-analysis of VEGF-C, VEGF-D and LVD in NSCLC. One meta-analysis has previously reported on VEGF-C and VEGFR3 and their association with survival in NSCLC [73]. Zhan et al. published the aggregated results of 8 studies reporting on VEGF-C and 4 studies allegedly reporting on VEGFR3 (The authors considered Flt1 to represent VEGFR3 –Flt1 is in fact equal to VEGFR1, while Flt4 is equal to VEGFR3) in 2009. They found the aggregated results of the studies reporting on VEGF-C and VEGFR3 to be non-significant in NSCLC [73]. Further, Wang et al. reviewed the aggregated results of 10 studies reporting on LVD and survival in NSCLC patients in 2012. They included multivariable adjusted results and in addition one study reporting VEGFR3 in tumor cell cytoplasm [16]. Since 2009 several studies on VEGF-C, VEGF-D, VEGFR3 and LVD have been published. This current meta-analysis correlates VEGF-C, VEGFR3 and high levels of LVD with nodal metastasis in NSCLC patients and identifies high VEGF-C (HR 1.57 95% CI: 1.34–1.84) and high levels of LVD (HR 1.84 95% CI: 1.18–2.87) as significant prognostic markers of poor survival.

### The VEGF-C/-D/R3 axis in relation to survival and lymphatic metastasis in NSCLC

VEGF-C, VEGF-D and their corresponding receptor VEGFR3 are well-known and strong lymphangiogenic markers [9,10,74]. In an extensive mapping of the VEGF-C/VEGFR3 axis in lung adenocarcinoma tumors, cell-lines and animal models Su. et al. found high expression of both VEGF-C and VEGFR3 to be correlated with nodal metastasis whereas expression was low for early-stage disease [11]. Further, NSCLC cell-lines over-expressing the VEGF-C/VEGFR3 axis showed increased migration, and, when introduced into xenograft models, more frequently formed lung metastases, compared to NSCLC cell-lines where VEGF-C/VEGFR3 signaling had been abrogated [11]. He et al. investigated the interaction between the lung cancer cell-line LNM35, which expresses high levels of VEGF-C, and lymphatic endothelial cells (LECs). They found VEGF-C to induce lymphatic vessel destabilization and enlargement of collecting lymphatic vessels, which further lead to passage of tumor clusters to sentinel lymph nodes [14]. In squamous cell carcinoma of the skin, Hirakawa et al. showed that VEGF-C over-expressing tumors maintained their lymphangiogenic profile after arrival in the sentinel lymph nodes [13]. Building on this, Liersch et al. found melanoma xenografts over-expressing VEGF-C to instigate lymphatic vascularization in the sentinel lymph nodes before the presence of metastatic cells could be detected [12]. These latter results are supported by a study on human surgical specimen (oral squamous cell carcinoma) demonstrating an increased number of high endothelial venules and lymphatic vessels without detecting metastatic cells [19]. However, in a recent study Nwogu et al. investigated the presence of nodal micro-metastases in pStage I and II NSCLC patients using conventional H&E, IHC and RT-PCR on resected lymph nodes. They found that 35/40, 33/40 and 16/40, respectively, were N0, indicating that conventional H&E and IHC might not be adequately sensitive to detect metastatic cells. They also demonstrated a strong correlation between the presence of nodal micro-metastases and the expression of VEGF-A, VEGF-C, VEGF-D and VEGFR3 in the lymph nodes [75]. Niki et al. isolated total RNA from 60 surgically resected lung adenocarcinomas of which 27 had lymph node metastasis and found only weak correlations between lymph node metastasis and VEGF-C. However, a high ratio VEGF-A,-B or -C to VEGF-D was associated with lymph node metastasis and the authors proposed that VEGF-D may have a regulatory role in tumor lymphangiogenesis [60]. Clearly, RNA expression does not necessarily translate to protein expression and it can be argued that a high ratio of VEGF-A, B or -C RNA to VEGF-D RNA simply represents over-expression of these molecules rather than VEGF-D having a regulatory role. The results of this meta-analysis indicate that VEGF-C is a prognostic factor for lymph node metastasis (HR 1.66 95% CI: 1.28–2.15) and survival (HR 1.57 95% CI: 1.34–1.84) in NSCLC patients, and further, that high expression of VEGFR3 is a prognostic factor for lymph node metastasis (HR 1.71 95% CI: 1.34–2.18)

### LVD in relation to survival and lymphatic metastasis in NSCLC

LVD describes the density of lymphatic vessels in the tumor micro environment [76]. In lymphovascular invasion (LVI) is an established adverse prognostic factor in NSCLC describing the presence of cancer cells within the lymphovascular space. LVI was recently reviewed by Mollberg et al. for stage I NSCLC [15]. There is controversy regarding the clinical implications of LVD in NSCLC. As of today, no consensus for the evaluation of LVD exists. This results in LVD being evaluated using several antibodies including D2-40, podoplanin, and VEGFR3 and by several techniques including the single most positive high-powered field (hot-spot), the invasive tumor front, the tumor center and combinations of these. Our systematic approach identified 12 studies reporting survival and 14 studies reporting associations with LNM in NSCLC patients. The studies varied in methodological approach with some evaluating the presence of LVD in hot-spots and some in random areas, either within the central tumor, in the invasive tumor front, a mix of different areas or location not given. This lead to a considerable difference in cut-offs used for high/low vessel count (summarized in Table 1). Unfortunately the number of studies utilizing the same methodology was too small to warrant sub-group analyses. Nevertheless, our meta-analysis identified high LVD-levels as a marker of poor prognosis (HR 1.84 95% CI 1.18–2.87) and LNM (HR 2.24 95% CIT 1.13–4.46) in NSCLC patients. Interestingly, Sun et al. found peritumoral LVD to be linked to survival while intratumoral LVD was not, suggesting this to be of interest for future studies [66]. This has an intuitive ring to it, as it seems logical that tumor cell migration to lymph nodes only can occur in functional lymphatic vessels mostly found in the tumor periphery and not in intratumoral lymphatic vessels that tend to be collapsed and non-functional. However, there is evidence suggesting that tumor cells may utilize the latter approach when entering the lymphatic system [76]. Renyi-Vamos et al. stratified patients based on low and high angiogenic activity in tumors, and found peritumoral LVD to significantly worsen the prognosis of those in the high angiogenic group [63]. In oral squamous carcinoma, LVD in sentinel nodes was elevated regardless of the presence of metastatic cancer cells, and correlated to tumor VEGF-C expression, suggesting an interplay between tumor and lymph node prior to the arrival of cancer cells [19]. In a recent meta-analysis, including 1044 breast cancer patients, LVD was correlated to LNM, but not to other known breast cancer traits [77]. Results of this meta-analysis coincide with the published literature of other cancer entities and suggests LVD as a prognostic marker for survival and LNM in NSCLC patients.

### Targeting lymphatic metastasis in the treatment of NSCLC

With the availability of small-molecule inhibitors and antibodies that could potentially target tumor lymphangiogenesis it seems appropriate that this treatment should be offered patients to whom it may prove beneficial. The challenge will be to select the correct patients. As pointed out in the xenograft model from He et al. timing seems to be of importance. In their model, no benefit of anti-VEGFR3 antibodies was observed when administered after the development of lymphangiogenic networks [14]. Evidently, results from murine models should be carefully evaluated before applied to actual human patients, but bad timing appears to be a feasible explanation for part of the moderate or lacking effects reported after a number of anti-angiogenic and also anti-lymphangiogenic approaches [78]. Interestingly, Tamura et al. have investigated serum VEGF-C levels of NSCLC patients with and without LNM [67]. They found high levels of VEGF-C to be associated with LNM with positive and negative predictive values of 70 and 77.3 respectively and that the addition of serum VEGF-A, serum matrix metalloproteinase 9 or CT-images further improved the diagnostic properties of the test [25,67,79]. These results were supported by the findings of Daly et al. in 2014 [80].

The results of this meta-analysis indicate that targeting lymphangiogenesis could prove beneficial for selected sub-groups of NSCLC patients. With timing being essential, theoretically, the best use of lymphangiogenic inhibitors could be in the adjuvant setting and for patients with low grade tumors in whom lymphatic node metastases have yet to develop. Obviously, the use of any drug in the adjuvant setting must be considered carefully as a proportion of patients have been cured of their cancer and will not benefit from further treatment. The studies linking serum levels of VEGF-C to nodal metastasis are especially interesting. These results indicate that NSCLC patients with no acknowledgeable LNM and high serum VEGF-C might be at risk and may be the patients who will benefit from anti-lymphaniogenic. According to the recent study by Daly et al. their group is setting up animal models with a plan to further elucidate their findings on serum-VEGF-C levels and LNM, hopefully culminating in a clinical trial; the results of these subsequent studies, will be very interesting [80].

During the past decade, cancer immunology has experienced a renaissance through the understanding of the immune check-points and the development of immune check-point inhibitors [81–83]. This important clinical research effort which initially succeeded in melanoma is now benefiting a wide host of other tumor groups [81–83]. Evidence indicates lymphangiogenic markers to mediate a profound impact on the immune system. Tumors expressing lymprhangiogenic factors are believed to alter the micro-environment in the lymph nodes, hence preparing them for the arrival and harboring of metastatic cells [12,13,19]. In fact, active lymphangiogenic processes may down-regulate immune responses and thus be partly responsible for the poor effect the immune system shows on most malignant tumors [84]. However, once the tumor associated lymphatic network is established, little is known of its role regarding the tumor immune responses. Tumor antigens must reach antigen presenting cells in order to induce a T-cell mediated immune response, and in this process the presence of, or interaction with, tumor-induced lymphatics may be of importance [84].

### Conclusions and implications for research

Our results indicate a connection between lymphangiogenic markers, LVD, LNM and survival in NSCLC. Nevertheless, it is apparent that lymphangiogenic factors cannot explain the full extent of LNM. Alternative pathways exist for cancer cells to recruit and invade lymphovascular structures, such as vessel co-option and vascular mimicry [85].

Further studies are warranted to evaluate the results of this meta-analysis and it would be highly interesting to see studies combining expressions of lymphangiogenic markers in primary tumor tissues and metastatic lymph nodes. In addition, we believe that studies investigating this intricate relationship between tumor-lymphangiogenesis and immunology will prove beneficial, not only for our understanding of these principles, but also for patients who could benefit from combined therapeutic approaches.

## Supporting Information

S1 PRISMA Checklist PRISMA Checklist. (DOC)
